# RETFound-enhanced community-based fundus disease screening: real-world evidence and decision curve analysis

**DOI:** 10.1038/s41746-024-01109-5

**Published:** 2024-04-30

**Authors:** Juzhao Zhang, Senlin Lin, Tianhao Cheng, Yi Xu, Lina Lu, Jiangnan He, Tao Yu, Yajun Peng, Yuejie Zhang, Haidong Zou, Yingyan Ma

**Affiliations:** 1 Shanghai Eye Disease Prevention & Treatment Center/ Shanghai Eye Hospital, School of Medicine, Tongji University, Shanghai, China; 2 National Clinical Research Center for Eye Disease, Shanghai, China; 3 Shanghai Engineering Center of Precise Diagnosis and Treatment of Eye Diseases, Shanghai, China; 4 Department of Ophthalmology, Shanghai General Hospital, Shanghai Jiao Tong University School of Medicine, Shanghai, China; 5 School of Computer Science, Shanghai Key Laboratory of Intelligent Information Processing, Fudan University, Shanghai, China

**Keywords:** Health care economics, Image processing, Eye diseases

## Abstract

Visual impairments and blindness are major public health concerns globally. Effective eye disease screening aided by artificial intelligence (AI) is a promising countermeasure, although it is challenged by practical constraints such as poor image quality in community screening. The recently developed ophthalmic foundation model RETFound has shown higher accuracy in retinal image recognition tasks. This study developed an RETFound-enhanced deep learning (DL) model for multiple-eye disease screening using real-world images from community screenings. Our results revealed that our DL model improved the sensitivity and specificity by over 15% compared with commercial models. Our model also shows better generalisation ability than AI models developed using traditional processes. Additionally, decision curve analysis underscores the higher net benefit of employing our model in both urban and rural settings in China. These findings indicate that the RETFound-enhanced DL model can achieve a higher net benefit in community-based screening, advocating its adoption in low- and middle-income countries to address global eye health challenges.

## Introduction

Eye health is a critical aspect of public health. The Global Burden of Disease Study revealed that in 2020, ~43 million individuals globally were blind, with approximately 295 million and 258 million individuals suffering from moderate to severe and mild visual impairments respectively^1^. Visual impairment not only significantly undermines visual function, thereby impacting quality of life^2^, but also lowers productivity and considerably increases all-cause mortality rates^3^. Previous studies have confirmed that effective eye disease screening can reduce the prevalence of blindness^4^. The development of diagnostic products for eye diseases assisted by artificial intelligence (AI) has matured in recent years^5^, with a growing number of such products receiving medical device approval and entering the market. Research indicates that the adoption of AI for eye disease screening can significantly alleviate the dependency on ophthalmologists and improve the cost-efficiency of screenings^6–8^. However, during community eye disease screening, the inability to perform pupil dilation, coupled with inferior image capture conditions, personnel, and environment compared with clinical settings, results in compromised image quality. The accuracy of conventional AI products developed for community eye disease screening significantly diminishes in comparison with the developmental phase^9,10^, failing to meet the screening needs of the community^11^. Similar concerns have been broadly observed in other medical domains, such as tumour imaging^12^. Therefore, improving the accuracy of AI in practical screening tasks is a pressing concern^13^.

On 13 September 2023, Zhou et al. published a research report wherein they employed Self-Supervised Learning (SSL) techniques on 1.6 million unlabelled fundus images to train a Vision Transformer (ViT), establishing a novel foundational model for retinal image recognition, RETFound^14^. This represents a fundamental shift from the preceding technological pathways. Traditional AI model development requires extensive labelled data^15^ and these models are often designed for specific tasks, exhibiting limited generalisation for various clinical applications. Conversely, RETFound was engineered to learn generalisable representations from unlabelled retinal images, thereby providing a foundation for rapid adaptation in multiple application realms. According to Zhou et al., RETFound can be rapidly deployed to downstream tasks through transfer learning, such as automatic diagnosis of diabetic retinopathy and glaucoma, prognostic forecasting of age-related macular degeneration, and systemic disease prediction. In various eye disease diagnostic tasks performed for internal validation on public datasets, its accuracy surpassed traditional models trained via supervised learning on ImageNet (SL-ImageNet) and proved superior to models entirely based on self-supervised learning on either ImageNet (SSL-ImageNet) or retinal images (SSL-Retinal)^14^. To what extent can the accuracy of community eye disease screening be enhanced by employing deep-learning (DL) models based on RETFound? What value can the RETFound framework potentially bring to community eye disease screening? The key questions concerning the extended application and promotion of the RETFound framework are yet to be answered.

For low-income and middle-income countries, the scarcity of health resources necessitates empirically validated evidence before the dissemination and application of new technologies to help formulate the most appropriate health policies. In this study, we developed a RETFound-enhanced DL model for multiple eye diseases using an image dataset from real-world community screening in Shanghai, China. The screening accuracy of the model was then compared with commercial models and traditional AI models to ascertain the degree to which the RETFound framework influences the screening accuracy. Furthermore, based on the prevalence of eye disease in urban and rural areas of China, a decision curve analysis (DCA) was applied to estimate and compare the net benefits of the different models.

## Results

### Performance on community-based eye disease screening

The test set incorporated in this study consisted of 1890 images, including 287 AMD, 337 DR, 151 PM, and 1115 normal fundus images. The proportion of images with blind eye disease in the test set exceeded the population prevalence. The comparative results are as follows (Table 1).

**Table 1:** Performance on community-based eye disease screening

Model Name	Evaluation metrics	AMD (*n* = 287)	DR (*n* = 337)	PM (*n* = 151)	Normal fundus (*n* = 1115)
RETFound-enhanced Model	Sensitivity	75.96 (218)	95.25 (321)	100 (151)	/
	Specificity	/	/	/	92.47 (1031)
Model S	Sensitivity	61.32 (176)	78.93 (266)	59.60 (90)	/
	Specificity	/	/	/	77.67 (866)
Model Y	Sensitivity	59.23 (170)	71.22 (240)	72.19 (109)	/
	Specificity	/	/	/	73.63 (821)

(1) There was a significant difference in the sensitivity to AMD among the RETFound model, Model S, and Model Y (*X*^2^ = 21.09, *p* < 0.001). Furthermore, post-hoc pairwise comparisons indicated that the sensitivity of the RETFound model was significantly higher than that of Model S (*X*^2^ = 14.28, *p* < 0.001) and Model Y (*X*^2^ = 18.33, *p* < 0.001). No significant differences were observed between the two commercial models (*X*^2^ = 0.61, *p* = 0.61).

(2) Regarding the sensitivity of DR, there was a significant difference among the RETFound model, Model S, and Model Y (*X*^2^ = 68.18, *p* < 0.001). Furthermore, post-hoc pairwise comparisons suggested that the sensitivity of the RETFound model was significantly superior to that of Models S (*X*^2^ = 39.92, *p* < 0.001) and Y (*X*^2^ = 69.76, *p* < 0.001). No significant difference is observed between the two commercial models (*X*^2^ = 5.36, *p* = 0.02, exceeding α′).

(3) For PM sensitivity, there was a significant difference among the RETFound model, Model S, and Model Y (Fisher’s *p* < 0.001). Moreover, post-hoc pairwise comparisons indicated that the sensitivity of the RETFound model was significantly higher than that of Models S (Fisher’s *p* < 0.001) and Y (Fisher’s *p* < 0.001). No significant difference is noted between the two commercial models (*X*^2^ = 5.32, *p* = 0.02, exceeding α′).

(4) For specificity, there is a significant difference among the RETFound model, Model S, and Model Y (*X*^2^ = 143.97, *p* < 0.001). Additionally, post-hoc pairwise comparisons revealed that the sensitivity of the RETFound model significantly outperformed that of Models S (*X*^2^ = 96.11, *p* < 0.001) and Y (*X*^2^ = 140.48, *p* < 0.001). No significant difference is identified between the two commercial models (*X*^2^ = 4.93, *p* = 0.03, exceeding α′).

### Net benefit in community-based eye disease screening

**Fig. 1:**
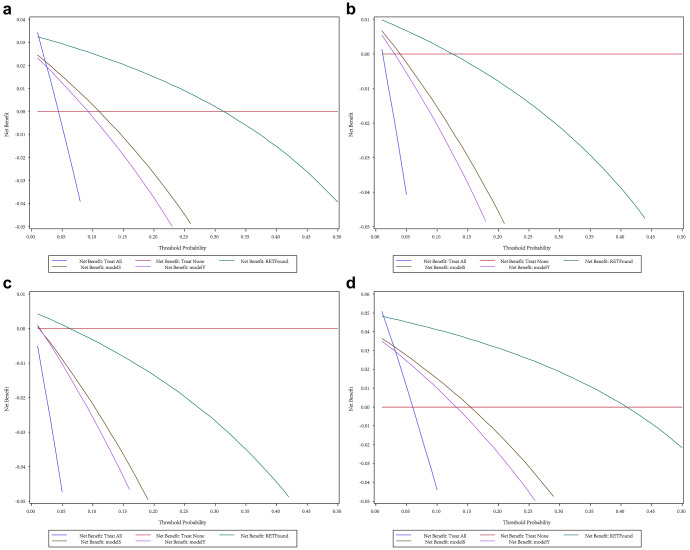
Comparison of using RETFound-enhanced model and commercial models in rural areas of China. **a** AMD single-disease screening. **b** DR single-disease screening. **c** PM single-disease screening. **d** multi-disease combined screening.

**Fig. 2:**
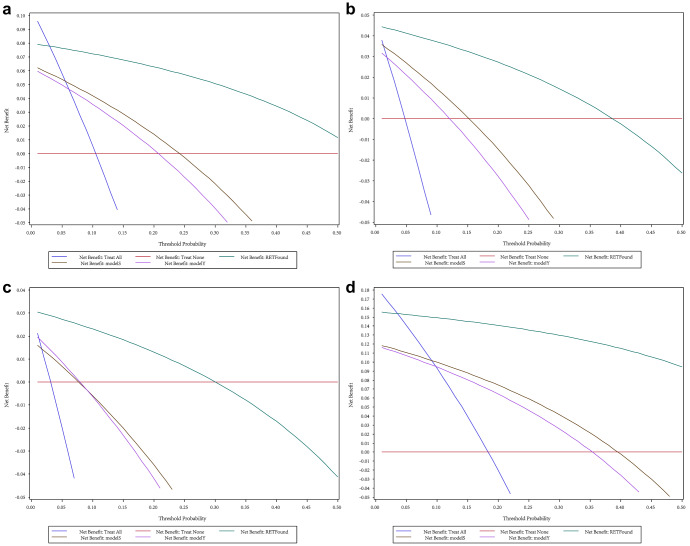
Comparison of using RETFound-enhanced model and commercial models in urban areas of China. **a** AMD single-disease screening. **b** DR single-disease screening. **c** PM single-disease screening. **d** multi-disease combined screening.

The DCA results indicated that in rural areas of China (Fig. 1) and urban areas of China (Fig. 2), whether for single-disease screening or multi-fundus disease screening, employing the RETFound-enhanced model can achieve higher net benefits than current commercial models. Specifically, in rural areas, the model exhibits maximal net benefit at probability thresholds ranging from 2% to 40%. In urban areas, optimal net benefit is achieved with thresholds between 4% and 71%. Although the two commercial models have advantages in terms of accuracy for various eye diseases, their net benefits for screening are relatively similar. Moreover, a comparison between Figs. 1 and 2 reveal that the net benefit of screening in urban areas surpasses that of screening in rural areas. This is attributed to the higher prevalence of diseases in urban areas, and the implementation of eye disease screening is anticipated to identify more patients.

### Comparison of the RETFound-enhanced model and CNNs

**Fig. 3:**
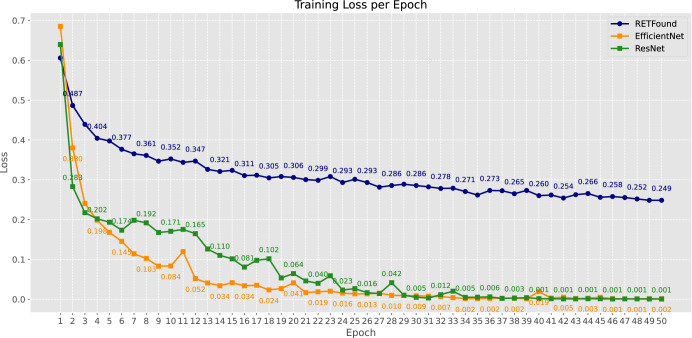
Comparison of model training loss over epochs. Each line represents the training loss for three distinct deep learning models across 50 epochs. The blue line corresponds to the RETFound-enhanced model, the orange line to the EfficientNet model, and the green line to the ResNet model.

As shown in Fig. 3, the convergence speeds of the models were similar, and the loss on the training set stabilised after 15 training cycles. Compared to the RETFound-enhanced model, the two CNN models exhibited a lower final loss in the training set.

**Fig. 4:**
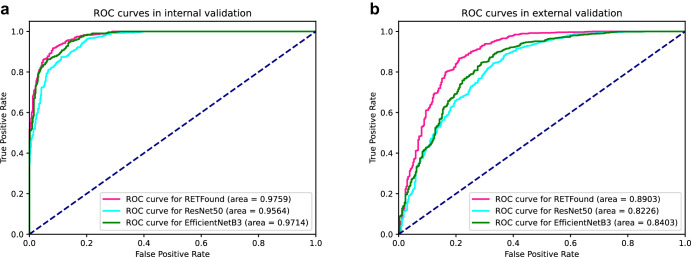
ROC curves of each model in internal and external validation. **a** internal validation. **b** external validation. ROC receiver operating characteristic curve.

The validation results for the three models are presented in Table 2 and Fig. 4. Both the RETFound-enhanced and traditional CNN models showed similar ability in identifying DR (from AUC 0.9564 to AUC 0.9759) using the fundus photographs from the internal validation set. The sensitivity of all models for recognising DR was higher than 90%, and the sensitivity of the RETFound-enhanced model was as high as 96.22%. Their performance was also good in recognising a normal fundus, and the specificity of all models was above 80%.

**Table 2:** Performance on DR classification

Model Name	Evaluation metrics	Internal validation		Youden’s index	External validation		Youden’s index
		DR (*n* = 502)	Normal fundus (*n* = 517)		DR (*n* = 337)	Normal fundus (*n* = 1115)	
RETFound	Sensitivity	96.22 (483)	/	0.7939	82.79 (279)	/	0.6127
	Specificity	/	83.17 (430)		/	78.48 (875)	
EfficientNetB3	Sensitivity	90.64 (455)	/	0.7826	67.95 (229)	/	0.5055
	Specificity	/	87.62 (453)		/	82.60 (921)	
ResNet50	Sensitivity	92.83 (466)	/	0.7871	61.72 (208)	/	0.5328
	Specificity	/	85.88 (444)		/	91.56 (1021)

However, in the external validation set, the CNN models exhibited a substantial drop in performance when compared with a new dataset with significantly different data distributions. By contrast, the RETFound-enhanced model was able to identify disease-related patterns (Supplementary Fig. 1) and demonstrated efficient performance when dealing with data that were different from the training set source. The AUROC and Youden’s indices for all three models have decreased, suggesting that during the generalisation process, the accuracy of all three models has been compromised. However, RETFound exhibits the smallest decrease, while the two CNN models show a similar level of decline. This indicates that the RETFound-enhanced model may have stronger generalisation and generic recognition capabilities than traditional healthcare AI approaches that train CNNs based on SL methods.

## Discussion

This is one of the first studies to evaluate the real-world value of the RETFound framework for eye disease screening. Using a foundation model pretrained via SSL to develop deep learning models for real-world community-based eye disease screening, our method demonstrated the highest sensitivity for the three main blinding retinal diseases. The results suggest that this SSL paradigm can be time-efficient and achieve higher net benefits than current commercial models and conventional SL-based CNNs.

**Fig. 5:**
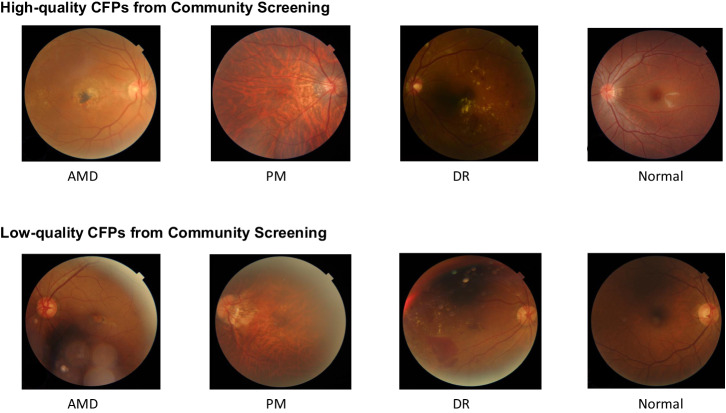
Display of typical images from the SDEDS image dataset. During community eye disease screenings, photographs are often taken by trained general practitioners or nurses under non-darkroom and non-dilated conditions, resulting in image quality that contrasts sharply with those taken by professional optometrists or ophthalmologists in ophthalmic clinical institutions under darkroom and dilated conditions. SDEDS Shanghai Digital Eye Diseases Screening Program, CFPs colourful fundus photographs, AMD age-related macular degeneration, PM pathological myopia, DR diabetic retinopathy.

China is one of the countries with the most severe vision impairment and blindness globally. In 2019, China reported 50.59 million individuals with moderate or severe vision impairment, and 8.69 million experiencing blindness^16^. The commercial models used as the control groups in this study have broad applications in China. However, a significant reduction in diagnostic accuracy compared to the R&D stage was observed upon implementation in real-world community eye disease screenings^9,10,17,18^. The decline in the accuracy of commercial models is primarily attributed to the image sets used during the training stage, which are typically derived from high-quality images obtained from clinical institutions^19–23^. However, community eye disease screening often fails to meet the requirements of clinical settings. Consequently, the images obtained during community eye disease screening are of inferior quality to those obtained from clinical institutions, resulting in heterogeneity between the working image sets and training sets of commercial models. This heterogeneity particularly affects the diagnostic accuracy for mild conditions. Mild conditions typically exhibit small and concealed lesions, necessitating high-quality images to clearly delineate the differences between lesions and surrounding areas; however, community imaging lacks such conditions, often resulting in under- or over-exposure and blurred details (Fig. 5), which in turn diminishes the detection capability of previous models for mild lesions during community screening (Supplementary Table 2). Moreover, the prevalence of low-quality images is primarily attributed to non-mydriatic conditions in community screenings. Advancements in non-mydriatic camera technology have the potential to significantly expand the scope of community screenings, from which our model stands to benefit considerably^24,25^.

Unlike CNNs, foundation models, such as RETFound, are pretrained on a large amount of unlabelled data and capture a wide range of data patterns through SSL. The foundation model is called “foundation” because it is trained on large amounts of data and allows us to finetune it for downstream tasks with minimal additional data. Prior to RETFound, there was no foundational model in the field of ophthalmology. Specifically, when the aim is to adjust and optimise models for a population in a certain community or a certain type of blinding eye disease, traditional medical AI methods utilise CNN models and pretrain them using ImageNet in a supervised manner. This often requires large amounts of training data and repetitive refinement to achieve satisfactory results for downstream tasks. In addition, CNN models may overfit the training data (i.e., extract features that are not relevant to the lesion), resulting in poor performance when adapted to new environments with different data characteristics. In this study, with only several thousand images, minimal GPU resources, and a shorter model training time, the RETFound framework swiftly adapted to our downstream tasks and exhibited good diagnostic accuracy (Supplementary Fig. 2). Additionally, it demonstrated enhanced generalisation capabilities when contrasted with models like EfficientNet and ResNet. Considering the diverse ethnic origins of the public databases used for model training and the SDEDS database employed for validation, this further substantiates the model’s broad applicability and generalisability. We believe that the paradigm of fine-tuning downstream tasks based on pretrained foundation models represents a key shift in medical AI research, providing an efficient alternative to the laborious, costly, and time-consuming data annotation process inherent in the traditional process of training CNNs based on SL methods. This paves the way for population- or region-specific AI model calibration, thereby creating personalised eye disease screening models, which is a unique advantage in ophthalmic applications.

The DCA results of this study indicated different net benefits between urban and rural areas in China when conducting community eye disease screening. This is partly attributable to the higher prevalence of diseases in urban areas, where more patients can be identified through screening. However, the deep learning model developed using the RETFound base model consistently exhibited higher net benefits than commercial models, whether in single-disease screening for AMD, PM, DR, or multi-disease combined screening. Therefore, screening accuracy is pivotal in this aspect. Previous studies by our team suggest that owing to the lower human resource costs in developing countries, the cost-effectiveness of utilising AI for community eye disease screening may not necessarily be better than employing remote screening technologies^26^. This study validated that the free, open-source RETFound model yields higher accuracy and net benefits in community eye disease screening than current commercial models. This holds significant value in promoting the utilisation of AI models in community eye disease screening in developing countries, including China. Further research can conduct health economic evaluations of the application of the RETFound framework model in the real world, which we believe will help to further supplement and validate the conclusions of this study.

This study has several notable advantages. First, we pioneered the demonstration that foundational models, such as RETFound, can accelerate the progress of AI in medicine by creating personalised models. In addition to reducing the likelihood of overfitting (which is common in CNNs) to improve generalisability, it also allows healthcare practitioners using AI to focus more on the functionality and application scenarios of AI models rather than on data collection or algorithm development. Second, it emphasises the net benefit as the primary outcome, surpassing the sole focus on screening diagnosis accuracy. This approach provides direct evidence of the practical application of the RETFound foundation model for eye disease screening. Third, all images used in this study were sourced from a long-term SDEDS project conducted in Shanghai, rather than clinical scenarios. This makes it a more suitable environment for evaluating the real-world accuracy of AI models.

This study has some limitations. First, it encompasses only two commonly encountered commercial models in China because of the unavailability of internationally recognised models, such as IDx-DR, OpthAI, RetinAI Medical, Retmarker, and Eyenuk. However, it is important to emphasise that our primary focus was not to conduct direct comparisons with commercial models. Instead, we focused on exploring the feasibility of developing automated screening tools based on foundational models using real-world and population-specific data. Second, the assessment of the accuracy and utility of the RETFound framework model in this study was based on the SDEDS Image Dataset without its actual implementation in the field. Nevertheless, our findings indicate that adopting the RETFound framework model can lead to substantial improvements in accuracy and net benefits. This finding strengthens the robustness of our conclusions.

In conclusion, the development of AI methods for eye disease screening using RETFound can yield net benefits that exceed those of commercial models and have better generalisability than traditional CNN models, while the development process is rapid. Thus, we recommend the application of the RETFound framework in real-world community screening for blinding eye diseases and encourage commercial companies to advance their intelligent eye disease screening products based on RETFound.

## Methods

The study was divided into three parts. We conducted a community eye disease screening program in Shanghai, China (Shanghai Digital Eye Disease Screening Program [SDEDS]). Since 2021, our team has retrospectively identified images of suspected eye diseases from past screenings and organised ophthalmology experts to conduct image readings for diagnosis (diagnostic criteria in Supplementary Table 1), gradually building a community eye disease screening image dataset (SDEDS dataset; Fig. 5). Currently, this dataset encompasses 17,249 images, including 1432 of age-related macular degeneration (AMD), 1682 of diabetic retinopathy (DR), 2485 of glaucoma, 748 of pathologic myopia (PM), 5334 of tessellated fundus, and 5568 of normal fundus. Image datasets continue to expand dynamically. First, we conducted a cross-sectional study. We developed a DL model enhanced by RETFound, based on transfer learning and the SDEDS dataset. We compared the accuracy of this model in multi-disease eye disease screening with that of two commercial models (anonymous models S and Y) that are widely used in China. The relevant results are derived from real-world operational databases and do not involve company participation, making them unsuitable for disclosing specific company names. Second, we combined the aforementioned accuracy and prevalence of eye diseases in urban and rural areas of China^4^ as parameters and constructed a hypothetical cohort of 100,000 individuals. The DCA technique was employed to evaluate the net benefit of implementing the RETFound-enhanced model for individual ocular disease screening in urban and rural areas of China. Third, we conducted a detailed comparison between the RETFound-enhanced DL model and traditional convolutional neural network (CNN) models trained via SL on ImageNet.

### Part one: construction and evaluation of RETFound-enhanced DL model

**Fig. 6:**
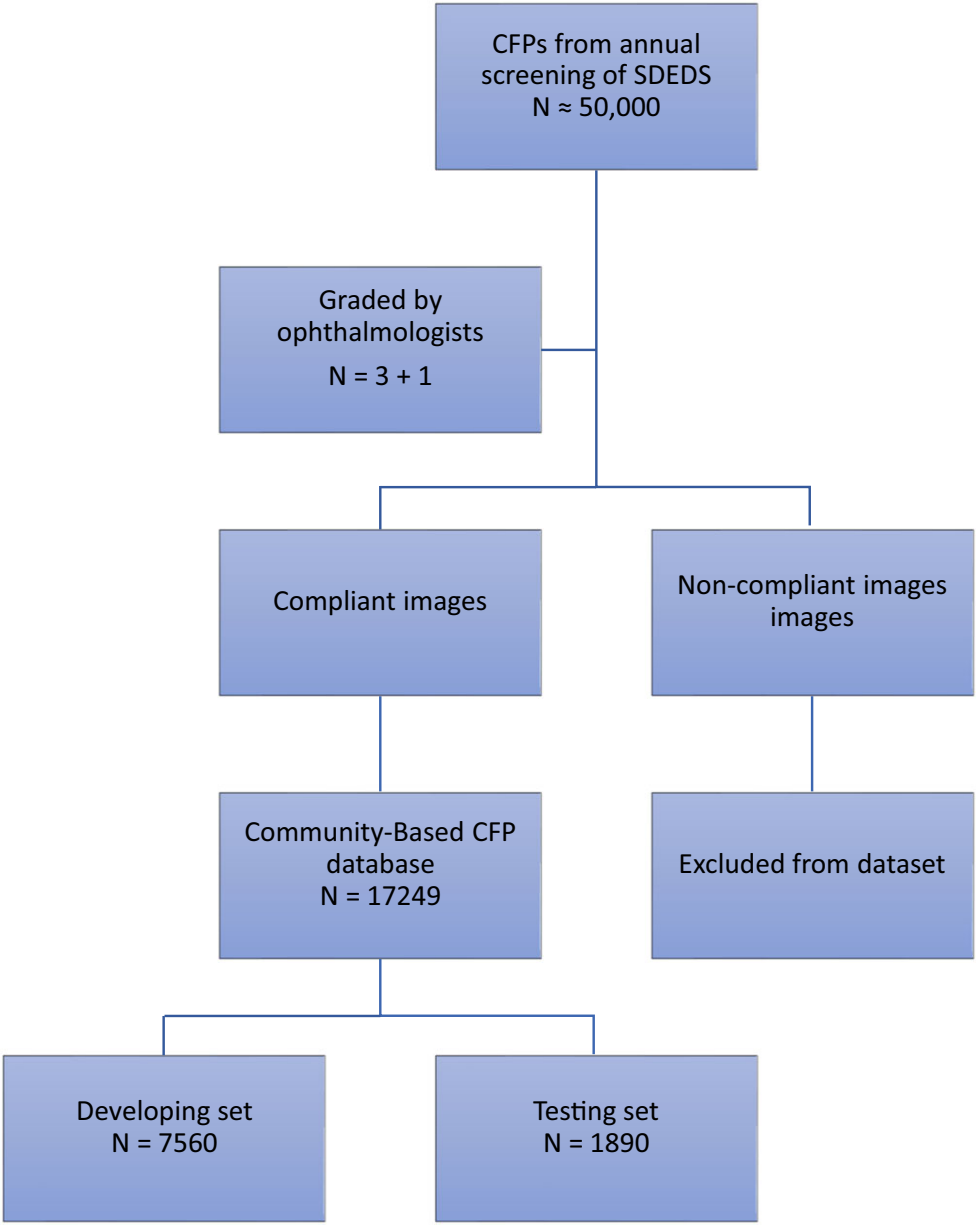
SDEDS image dataset inclusion and building workflow. The final selection of all fundus photographs underwent a thorough quality review and was meticulously annotated.

The data used in this study were sourced from the SDEDS dataset (Fig. 6). Each fundus image was independently classified and annotated by three ophthalmologists. In cases of discrepancy, collective deliberation involving ophthalmologists and a senior retinal specialist was convened to determine the final diagnoses. All images were re-evaluated based on the following criteria: the retinal fovea was not fully visible or obscured in over 50% of the total area, blurriness, severe artefacts, low contrast, uneven lighting, and excessive reflectance. Eventually, from the pool of images conforming to the criteria and upon expert review, a random assortment of 7560 images encompassing DR, PM, AMD, and no-eye disease was used as the development dataset. An additional 1890 images, including DR, PM, AMD, and no-eye disease images, were randomly chosen to constitute the test dataset.

Our study adhered to the principles of the Declaration of Helsinki and was approved by the ethics committee of Shanghai Eye Diseases Prevention and Treatment Centre. This study exclusively utilised retrospective data, with all images undergoing irreversible anonymisation and no active patient engagement, so informed consent was deemed not applicable. No commercial interest was implicated in the design or execution of this study.

**Fig. 7:**
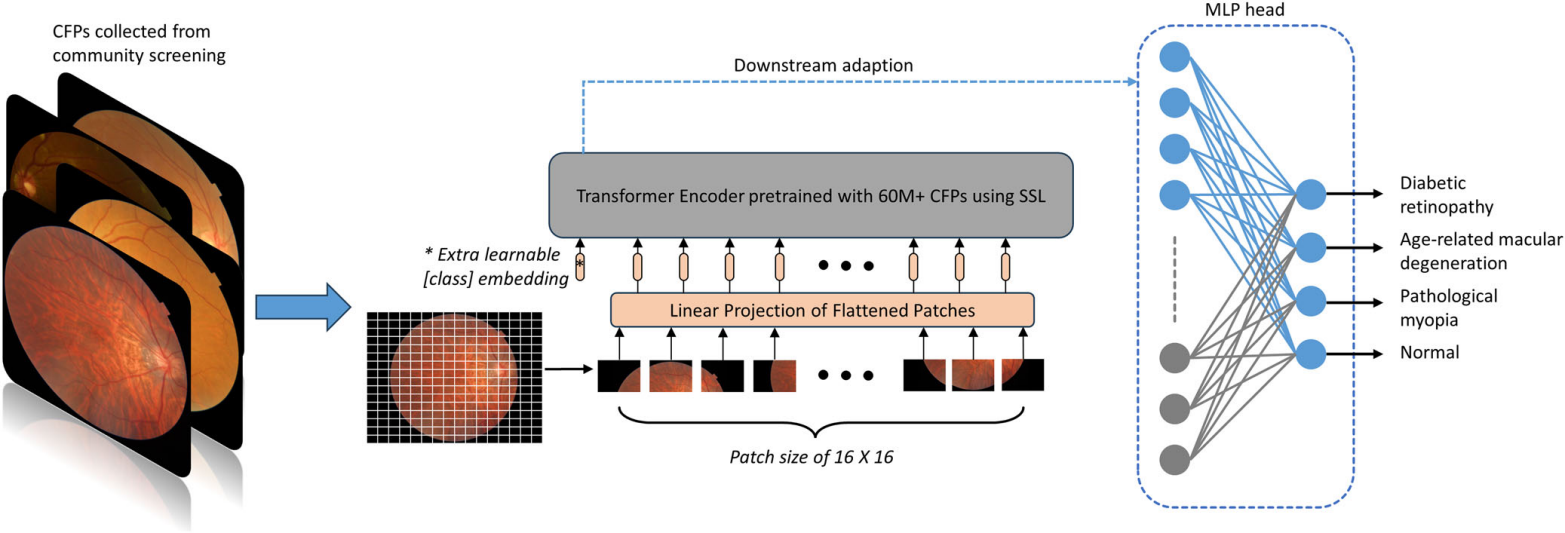
Overview of the RETFound-enhanced DL model for community-based fundus diseases screening. The model consists of a pretrained transformer encoder and a MLP head.

The construction process of the RETFound-enhanced eye disease screening model is illustrated in Fig. 7. We employed the encoder component of RETFound, which utilises the ViT-large architecture^27^ and features 24 transformer blocks with an embedding vector size of 1024. The encoder accepts unmasked patches (with a patch size of 16 × 16) as input and projects them into a feature vector with a size of 1024. The 24 transformer blocks, which comprise multiheaded self-attention and a multilayer perceptron, process these feature vectors to generate high-level features. Subsequently, these high-level features are input into a multilayer perceptron (MLP) head, which produces the final predicted categories.

Of the 7560 images in the development dataset, 6599 were designated as the training set, including 1002 images of AMD, 1177 of DR, 523 of PM, and 3897 of normal fundus, and 941 were allocated to the validation set, encompassing 143 images of AMD, 168 of DR, 74 of PM, and 556 of normal fundus. All images were resized to 256 × 256 pixels using cubic interpolation. All images underwent the same data augmentation procedures as those during model training, including random cropping (with a cropping range of 20% to 100% of the entire image), followed by resizing the cropped image blocks to 224 × 224, random horizontal flipping, and image normalisation. The training objective was to generate classification outputs congruent with the labels. In this study, four categories were used: DR, PM, AMD, and normal fundus. The training was conducted using four NVIDIA GeForce RTX 2080 Ti GPUs, with CUDA version 11.1, powered by an Intel(R) Xeon(R) Platinum 8255C CPU @ 2.50 GHz, in the Ubuntu 18.04 system environment with 86 GB of memory. The batch size was set to 16. A total of 50 training epochs were set, with the initial 10 epochs designated for the warm-up phase (the learning rate gradually increased from 0 to 5 × 10^−4^), followed by cosine annealing scheduling (the learning rate gradually descended from 5 × 10^−4^ to 1 × 10^−6^). After each epoch, the model was evaluated using a validation set. The model weights with the highest AUROC in the validation set were preserved as model checkpoints for testing and DCA.

After the training phase, we determined the precise diagnostic sensitivity and specificity of our model using a test set. Subsequently, we employed the *χ*^2^ test (or Fisher’s Exact test) and post-hoc pairwise comparisons (via the Bonferroni method, *α*′=0.05/3) to compare the sensitivity and specificity of our RETFound-enhanced model with those of two commercial models.

### Part two: decision curve analysis

The primary outcome was the net benefit. The metrics commonly used to evaluate prediction models include sensitivity and specificity. However, these measures do not provide insight into the practical applicability of the model. The thresholds for sensitivity and specificity necessary to endorse its clinical use are ambiguous. Likewise, the level of miscalibration deterring the use of a prediction model or the criteria for selecting between two models—one with superior calibration and the other with enhanced discrimination—remain undefined. Therefore, decision curves have emerged as a prevalent tool for assessing the clinical utility of prediction models by analysing their net benefits^28^.

The net benefit is expressed as

1 $${Net}\,{benefit}=\frac{{TruePositiveCount}}{n}-\frac{{P}_{t}}{1-{P}_{t}}\times \frac{{FalsePositiveCount}}{n}$$

where *P*_t_ represents the probability threshold at which the expected benefit of engaging in subsequent therapy (or further testing) balances the expected benefit of avoidance. In the context of diagnostic testing, doctors are required to discern the precise risk level that merits further intervention. For instance, some may consider a 10% risk of blinding diseases warranting further therapy after an adverse reaction assessment, whereas others may suggest a 20% risk criterion with a more cautious stance. This risk cutoff point was characterised as the probability threshold in the decision curve analysis. One model may be favoured over another if its net benefit exceeds that of the other models at the selected threshold probability^28^.

We used DCA to compare the net benefits of applying our RETFound-enhanced DL model and two commercial models to real-world scenarios. DCA is a statistical technique for evaluating the clinical outcomes of models and tests. Traditional accuracy metrics, such as the AUROC or Brier score, disregard situational considerations. DCA assesses the net benefit of a model against the two standard strategies of treating all patients or treating none.

### Part three: comparison with CNN baselines

CNNs have been the standard for automated medical image diagnosis over the last decade^29^. Transformers, particularly ViTs, have recently gained prominence. To make further comparisons with traditional CNN models, we designed the following task:

We chose ResNet50 and EfficientNetB3, pretrained using SL on ImageNet-21k, as representatives. Three commonly used public datasets (MESSIDOR-2, APTOS-2019, and IDRiD) were selected. The automatic diagnosis of DR based on fundus images was one of the earliest applications of DL in ophthalmology. Relevant public fundus image datasets are numerous, well recognised, and of excellent quality. Therefore, we focused on the DR to compare our RETFound-enhanced model with the two CNN models. Basic information about the data is presented in Table 3.

**Table 3:** Characteristic of three public datasets of DR fundus images

Dataset	DR	Normal	Total
MESSIDOR-2	731	1017	1748
APTOS-2019	1496	1433	2929
IDRiD	279	134	413
Total	2506	2584	5090

Eighty percent of these public datasets were randomly selected to train the three models, and 20% were used for internal validation. The training process was the same as Part One, and 50 epochs were performed. The model parameters with the highest accuracy in the internal validation set were saved and tested using the DR part of the test dataset in Part One for external validation.

### Reporting summary

Further information on research design is available in the Nature Research Reporting Summary linked to this article.

## Supplementary information

Supplementary Information file

Reporting Summary

## Data Availability

The export of human-related data is governed by the Ministry of Science and Technology of China (MOST) in accordance with the Regulations of the People’s Republic of China on Administration of Human Genetic Resources (State Council No.717). A request for the non-profit use of the fundus images in the SDEDS should be sent to corresponding author Yingyan Ma.

## Code availability

The code of RETFound model for fine-tuning own model is available at https://github.com/rmaphoh/RETFound_MAE. The code for finetuning our model is available at https://github.com/Akemimadokami/DL-Model-for-Community-based-Fundus-Disease-Screening. The analytical procedure for DCA was sourced from the SAS macro available at https://raw.githubusercontent.com/ddsjoberg/dca.sas/main/dca.sas.
