# Supplementary material for: RETFound-enhanced community-based fundus disease screening: real-world evidence and decision curve analysis
Source: NPJ Digit Med. 2024 Apr 30;7:108. doi: 10.1038/s41746-024-01109-5 (PMC11063045; doi:10.1038/s41746-024-01109-5)

Supplementary Table 1. Grading and definition of AMD, PM, Tessellated Fundus, DR, and normal fundus.

**DR[1]**	**Mild NPDR**	Microaneurysms only
	**Moderate NPDR**	More than microaneurysms but less than severe NPDR
	**Severe NPDR**	Any of the following: ≥20 intraretinal hemorrhages in each of 4 quadrants; definite venous beading in ≥2 quadrants; or prominent RMA in ≥1 quadrant and no PDR
	**PDR**	≥1 of: neovascularization, VH, or PRH
**AMD[2,3,4]**	**Early**	Any soft drusen (distinct or indistinct) and pigmentary abnormalities, or large soft drusen 125 µm or more in diameter with a large drusen area (>500 µm diameter circle) or large soft indistinct drusen in the absence of signs of late–stage disease
	**Geographic atrophy**	Presence of geographic atrophy or pigment epithelial detachment
	**Neovascular AMD**	Subretinal haemorrhage or visible subretinal new vessel, or subretinal fibrous scar or laser treatment scar
**PM[5]**	**A2**	Diffuse chorioretinal atrophy
	**A3**	Patchy chorioretinal atrophy
	**A4**	Macular atrophy
	**Choroidal neovascularization**	Lacquer cracks or myopic choroidal neovascularization
	**Myopic traction maculopathy**	Vitreomacular traction, Macular hole
**Tessellated Fundus[5]**	**A1**	Tessellated Fundus
**Normal**	\	No apparent abnormalities detected in the color fundus photographs

Source:

1. Wilkinson CP, Ferris FL, 3rd, Klein RE, Lee PP, Agardh CD, Davis M, et al; Global Diabetic Retinopathy Project Group. Proposed international clinical diabetic retinopathy and diabetic macular edema disease severity scales. Ophthalmology 2003;110:1677-1682
2. Klein R, Davis MD, Magli YL, et al. The Wisconsin age-related maculopathy grading system. Ophthalmology 1991; 98: 1128-34.
3. Bird AC, Bressler NM, Bressler SB, et al. An international classification and grading system for age-related maculopathy and age-related macular degeneration. The International ARM Epidemiological Study Group. Surv Ophthalmol 1995; 39: 367-74.
4. Seddon JM, Sharma S, Adelman RA. Evaluation of the clinical age-related maculopathy staging system. Ophthalmology 2006; 113: 260-6.
5. Kyoko OM, Ryo K, Jost BJ, et al. International Photographic Classification and Grading System for Myopic Maculopathy, American Journal of Ophthalmology, Volume 159, Issue 5, 2015, Pages 877-883.

Supplementary Table 2. Sensitivity Overview of Three AI Models for different DR

	Mild DR (n=72)	Moderate DR and above (n=265)	X^2	P value
RETFound	83.33 (60)	98.49 (261)	28.76	<0.001
Model S	59.72 (43)	84.15 (223)	20.32	<0.001
Model Y	62.50 (45)	73.58 (195)	3.39	0.065

The sensitivity of the RETFound model significantly surpasses that of the two commercial models. For mild DR, the sensitivity of all three models is inferior compared to their performance on moderate DR and above.

（1）For mild DR, there is a significant difference among the RETFound model, Model S, and Model Y (X^2=11.12， p=0.004). Additionally, post-hoc tests reveal that the sensitivity of the RETFound model significantly outstrips Model S (X^2=9.85， p=0.002) and Model Y (X^2=7.91， p=0.005). No significant difference is identified between the two commercial models (X^2=0.12， p=0.73).

（2）For moderate DR and above, there is a significant difference among the RETFound model, Model S, and Model Y (X^2=66.46， p<0.001). Additionally, post-hoc tests reveal that the sensitivity of the RETFound model significantly outstrips Model S (X^2=34.37， p<0.001) and Model Y (X^2=68.42， p<0.001). No significant difference is identified between the two commercial models (X^2=8.88， p=0.003).

Supplementary Figure 1. Qualitative results of the RETFound-enhanced model. Heatmap highlighting the areas that contribute to the classification of the RETFound-enhanced model in the downstream tasks. Red color indicates high contribution. The well-defined pathologies of ocular diseases are identified and used for classification.

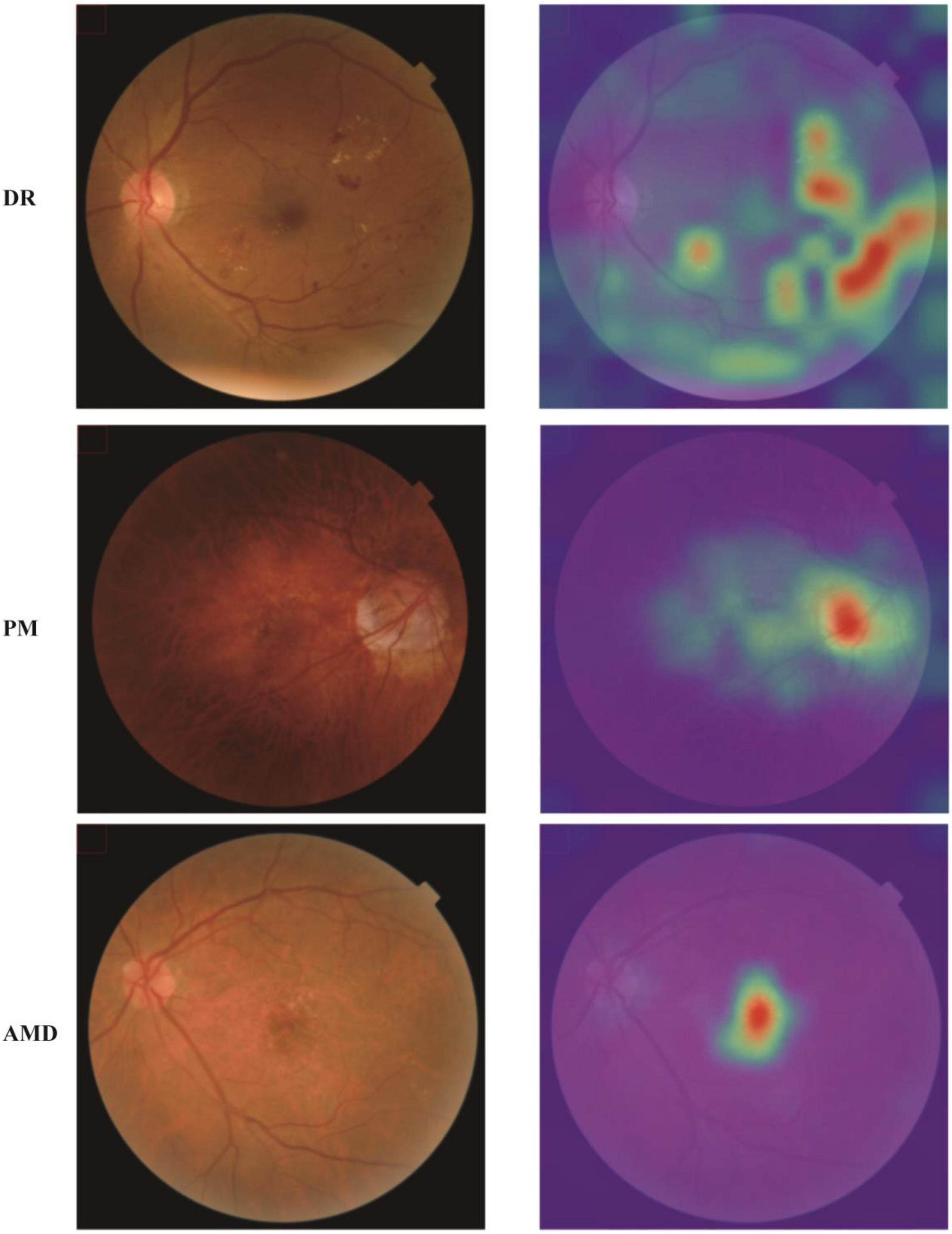

To ensure explainability, we used Grad-CAM specified for Transformer-based network. The principle of visualizing the output of ViT with Grad-CAM involves utilizing the outputs and gradients of the last attention block of ViT. It calculates the contribution of each token to the classification result, and then maps these contributions back to the spatial positions in the original image, forming a heatmap. As a result, it visualizes the areas of input images that lead to a certain classification.

Supplementary Figure 2. The adaptation process of RETFound-enhanced model. Training loss curve represents the loss function computed over the training dataset as the model learns during the training process. Validation accuracy curve shows the model's accuracy on a separate validation dataset that is not used for training.

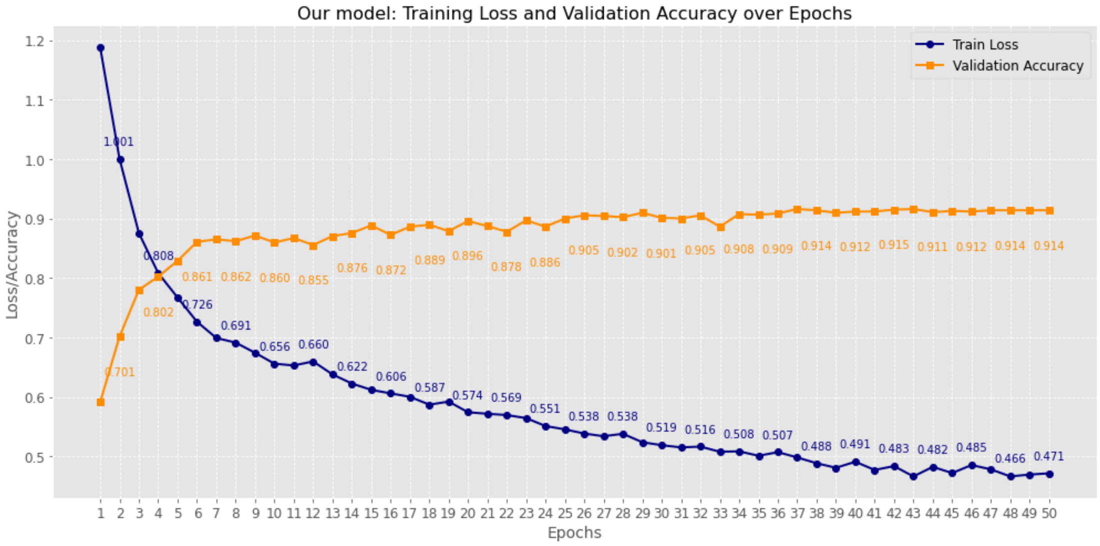

Supplement: Supplementary file 1 — Supplementary Information file [file 41746_2024_1109_MOESM1_ESM.pdf]
