# Supplementary material for: RETFound-enhanced community-based fundus disease screening: real-world evidence and decision curve analysis
Source: NPJ Digit Med. 2024 Apr 30;7:108. doi: 10.1038/s41746-024-01109-5 (PMC11063045; doi:10.1038/s41746-024-01109-5)
Supplement: Supplementary file 2 — Reporting Summary [file 41746_2024_1109_MOESM2_ESM.pdf]

nature portfolio

Corresponding author(s): Yingyan Ma, Haidong Zou, Yuejie Zhang

Last updated by author(s): 2024/03/25

Reporting Summary

Nature Portfolio wishes to improve the reproducibility of the work that we publish. This form provides structure for consistency and transparency in reporting. For further information on Nature Portfolio policies, see our Editorial Policies and the Editorial Policy Checklist.

Statistics

For all statistical analyses, confirm that the following items are present in the figure legend, table legend, main text, or Methods section.

n/a	Confirmed	
☐	☒	The exact sample size (*n*) for each experimental group/condition, given as a discrete number and unit of measurement
☐	☒	A statement on whether measurements were taken from distinct samples or whether the same sample was measured repeatedly
☐	☒	The statistical test(s) used AND whether they are one- or two-sided<br>*Only common tests should be described solely by name; describe more complex techniques in the Methods section.*
☒	☐	A description of all covariates tested
☒	☐	A description of any assumptions or corrections, such as tests of normality and adjustment for multiple comparisons
☐	☒	A full description of the statistical parameters including central tendency (e.g. means) or other basic estimates (e.g. regression coefficient) AND variation (e.g. standard deviation) or associated estimates of uncertainty (e.g. confidence intervals)
☐	☒	For null hypothesis testing, the test statistic (e.g. *F*, *t*, *r*) with confidence intervals, effect sizes, degrees of freedom and *P* value noted<br>*Give P values as exact values whenever suitable.*
☒	☐	For Bayesian analysis, information on the choice of priors and Markov chain Monte Carlo settings
☒	☐	For hierarchical and complex designs, identification of the appropriate level for tests and full reporting of outcomes
☒	☐	Estimates of effect sizes (e.g. Cohen's *d*, Pearson's *r*), indicating how they were calculated

Our web collection on statistics for biologists contains articles on many of the points above.

Software and code

Policy information about availability of computer code

Data collection	The code of RETFound model for fine-tuning own model is available at https://github.com/rmaphoh/RETFound_MAE. The code for finetuning our model is available at https://github.com/Akemimadokami/DL-Model-for-Community-based-Fundus-Disease-Screening. The analytical procedure for DCA was sourced from the SAS macro available at https://raw.githubusercontent.com/ddsjoberg/dca.sas/main/dca.sas.
Data analysis	Data was analysed with Python v3.6 (https://www.python.org/), NumPy v1.19.5 (https://github.com/numpy/numpy), SciPy v1.5.4 (https://www.scipy.org/), seaborn v0.12.0 (https://github.com/mwaskom/seaborn), Matplotlib v3.6.1 (https://github.com/matplotlib/matplotlib), pandas v1.5.0 (https://github.com/pandas-dev/pandas), Scikit-Learn v1.1.3 (https://scikit-learn.org/stable), Pillow v9.2.0 (https://pypi.org/project/Pillow).

For manuscripts utilizing custom algorithms or software that are central to the research but not yet described in published literature, software must be made available to editors and reviewers. We strongly encourage code deposition in a community repository (e.g. GitHub). See the Nature Portfolio guidelines for submitting code & software for further information.

Data

Policy information about availability of data

All manuscripts must include a data availability statement. This statement should provide the following information, where applicable:

- Accession codes, unique identifiers, or web links for publicly available datasets
- A description of any restrictions on data availability
- For clinical datasets or third party data, please ensure that the statement adheres to our policy

The export of human-related data is governed by the Ministry of Science and Technology of China (MOST) in accordance with the Regulations of the People's Republic of China on Administration of Human Genetic Resources (State Council No.717). Request for the non-profit use of the fundus images in the SDEDS should be sent to corresponding author Yingyan Ma.

Research involving human participants, their data, or biological material

Policy information about studies with human participants or human data. See also policy information about sex, gender (identity/presentation), and sexual orientation and race, ethnicity and racism.

Reporting on sex and gender	Experiments were conducted both on female and male. Since all of the data was collected retrospectively in a de-identified way, we do not have access to individual age or gender information for each image. We have conducted a statistical analysis on the demographic information of the image library for the year 2021 to understand the distribution of gender and age. Based on our statistical findings, males account for 43.88% of the image library, while females account for 56.12%. The age distribution has a mean value of 69.15 and standard deviation of 7.77.
Reporting on race, ethnicity, or other socially relevant groupings	The ethnicity are solely Chinese, since experiments were conducted on permanent resident population in Shanghai, China. SDEDS includes various imaging devices, such as DRI OCT Triton (Topcon), TRC-50DX (Topcon), TRC-NW400 (Topcon), CR-2 (Canon), VISUCAM 224 (Zeiss), and VISUCAM 524 (Zeiss).
Population characteristics	Experiments were conducted on permanent resident population in Shanghai, China who have been screened in our community eye disease screening program between 2012 and 2022. Only de-identified retrospective data was used.
Recruitment	All permanent resident population in Shanghai, China who have been screened in our community eye disease screening program between 2012 and 2022.
Ethics oversight	The study protocol was approved by Ethics Committee of Shanghai Eye Disease Prevention and Treatment Center.

Note that full information on the approval of the study protocol must also be provided in the manuscript.

Field-specific reporting

Please select the one below that is the best fit for your research. If you are not sure, read the appropriate sections before making your selection.

☒ Life sciences ☐ Behavioural & social sciences ☐ Ecological, evolutionary & environmental sciences

For a reference copy of the document with all sections, see nature.com/documents/nr-reporting-summary-flat.pdf

Life sciences study design

All studies must disclose on these points even when the disclosure is negative.

Sample size	Data employed in this investigation were sourced from the previously mentioned SDEDS Image Dataset. A random assortment of 7,560 images encompassing DR, PM, AMD, and no eye diseases were used as the development dataset. An additional 1,890 images inclusive of DR, PM, AMD, and no eye diseases were randomly chosen to constitute the test dataset.
Data exclusions	Data failed meet quality criteria mentioned in the Method part were excluded.
Replication	All patients were randomly selected and were not correlated in any way.
Randomization	The training/validation/testing data for downstream tasks were randomly splitted in ratio of 70%:10%:20%.
Blinding	All ophthalmologists who labeled the fundus images were blinded to the group group allocation and analysis. The investigators were not blinded to group allocation and analysis, because blinding was not relevant to our study due to the inherently blinded nature of test procedures using deep learning algorithm.

Reporting for specific materials, systems and methods

We require information from authors about some types of materials, experimental systems and methods used in many studies. Here, indicate whether each material, system or method listed is relevant to your study. If you are not sure if a list item applies to your research, read the appropriate section before selecting a response.

Materials & experimental systems

n/a	Involved in the study
☒	☐ Antibodies
☒	☐ Eukaryotic cell lines
☒	☐ Palaeontology and archaeology
☒	☐ Animals and other organisms
☒	☐ Clinical data
☒	☐ Dual use research of concern
☒	☐ Plants

Methods

n/a	Involved in the study
☒	☐ ChIP-seq
☒	☐ Flow cytometry
☒	☐ MRI-based neuroimaging

Plants

Seed stocks	not applicable
Novel plant genotypes	not applicable
Authentication	not applicable
